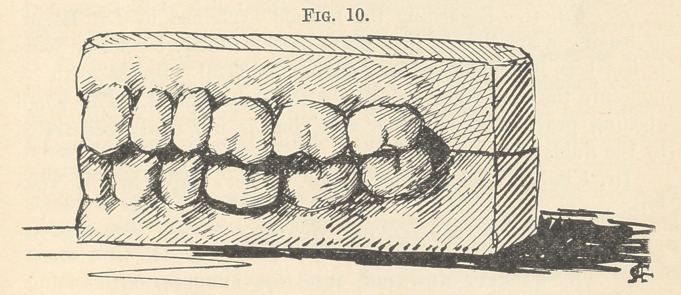# The New York Institute of Stomatology

**Published:** 1902-05

**Authors:** 

**Affiliations:** The New York Institute of Stomatology


### Reports of Society Meetings.

### THE NEW YORK INSTITUTE OF STOMATOLOGY.

A meeting of the Institute was held at the office of Dr. Chas. O. Kimball, No. 27 West Thirty-eighth Street, New York, on Tuesday evening, January 7, 1902, the President, Dr. J. Morgan Howe, in the chair.

The minutes of the last meeting were read and approved.

### COMMUNICATIONS ON THEORY AND PRACTICE.

*Dr. E. A. Bogue.*—In view of the difficulty of making approximal amalgam fillings the exact shape of the portion of tooth they are intended to replace, I have for several years adopted a method of insertion which my friend, Dr. F. Milton Smith, has encouraged me to present at this meeting.

In the first place the space between the natural teeth is much wider at the gum than at the point or line where the teeth are in contact. (Fig. 1.) In order to insert my amalgam fillings with the least possible delay and the greatest possible accuracy I have had constructed a number of steel blades like the end of a pen-

Fig. 1.

knife blade or small lancet. These lancet ends are like the model herewith presented. (Fig. 2.) In form, thicker at the back and

Fig. 2.

also at the heel, so that when the blade lies between two molar teeth, for example, with the butt end towards the lingual side and the thinner end towards the cheek, the edge towards the grinding surfaces of the teeth, it will just about fill the natural space existing between two adult molars. Not wishing always to cut away the grinding edges of teeth, or even to cut into them, I first wedge the teeth apart gently, using for this purpose the so-called grass line, which was exhibited and described by Dr. Davenport a year or two ago. A loop of this grass line (or twisted silk, as it is in reality) is drawn between the teeth from the buccal side towards the tongue by means of a floss-silk, which has been passed between the teeth and engaged in the loop, which is then drawn through between the teeth. (Fig. 3.) One end of the grass line is then passed through the loop on the lingual side (Fig. 4), after which the two ends are tied in three single knots on the buccal side of the teeth, drawing the double loop tightly upward towards the points of contact of the two teeth which are to be separated. This leaves a double loop of twisted silk between the teeth (Fig. 5), which gradually spreads with the moisture of the mouth and separates the teeth without falling out, hurting the gum, or causing much annoyance to the patient. This was my

Fig. 3.

Fig. 4.

Fig. 5.

first step in the present instance. Having obtained the necessary room, I placed the rubber dam with a clamp over the second molar and stretched it forward over tooth after tooth until the dam covered all the lower teeth on this side as far as the lateral incisor. The edges of the dam being turned towards the gum by means of a floss-silk slightly soaped so that it would not stick to the dam, I placed a separator over the molar and the bicuspid which was to be filled. The cavity was a large posterior approximal, having a pretty wide semilunar opening towards the tongue and a similar one towards the cheek. Using warm alcohol first and cocaine and carbolic acid afterwards to allay sensitiveness, I excavated and shaped my cavity in such manner as to admit of the insertion of my filling from the buccal side. When all was ready my assistant weighed out my amalgam fillings with the necessary amount of mercury, while I arranged the matrix for the filling in this fashion: I first put my lancet blade between the teeth, with the point towards the cheek and the butt towards the tongue. I next took a metal polishing strip with a roll at the end (Fig. 6), such as we used to find ready made in dental depots.

FIG. 6.

Placing this between the teeth, just in front of my lancet blade, I drew it gently towards me until the roll on the end of the polishing strip touched the head of the lancet blade, holding the latter in position. I then took a small piece of thin steel, previously cut out with scissors (Fig. 7), and laid this between the polishing

FIG. 7.

strip and the tooth to be filled, practically covering the whole cavity and somewhat more with the steel. (Fig. 8, *A*.) Now, by drawing firmly on the polishing strip I had a curved matrix towards the lingual side of the tooth, the matrix was held closely in position at the neck of the tooth by the lancet blade, which was thick at that point, and farther up towards the grinding surface the lancet blade, polishing strip, and steel were sufficiently loose to be pushed back from the cavity by the excess of amalgam as I placed it in position. My amalgam, having been mixed and made into small blocks, was handed to me, and piece by piece was malleted to its place, a wad of tissue-paper being placed between the instrument point and the amalgam. When my cavity was full the polishing strip was turned towards the front of the mouth, and drawn tightly against the tooth, the steel strip being between it and the amalgam. (Fig. 8, *B*.) This brought the amalgam into a nearly cylindrical form, leaving a little surplus towards the upper part of the cavity, which was near the grinding end of the tooth. I cut away with Dr. Kimball's oblique lancets what little surplus exuded. I absorbed, with freshly annealed soft gold cylinders, all surplus mercury that I could and then removed my matrices in the following order. The lancet blade was first pushed backward into the mouth; the polishing strip was next gently removed, taking care not to disturb the thin steel that lay against the amalgam. After this the thin steel was removed, and then with a thin silk ribbon, or very thin cotton tape, I was able to polish this filling into shape, removing all overhanging edges, and finding myself with a contour filling that completely restored the original shape and rounded tuberosity of the tooth, and all done with a certainty and despatch as agreeable to the operator as it must be gratifying to the patient.

By absorbing the mercury as I did, my filling was sufficiently set to maintain its form perfectly, though no further precautions were taken to protect it other than to counsel the patient to eat upon the other side for the next two meals.

I have another matter to present, in which Dr. Davenport encourages me. It is a method that I have used for over thirty years in cases where the teeth were greatly broken down.

The model which I hold in my hand represents a case that presented a few days since, where the entire crown had broken off, leaving the roots filled and in healthful condition. (Fig. 9.) I drilled into each of these lower molar roots nearly one-quarter of an inch and inserted a heavy gold screw into each drill-hole, cutting the screws off about on a level with the adjoining teeth; a platinum lined gold ring (thickness 32-gauge) was then made of just the circumference of the roots (a thin copper wire having been passed around the roots and twisted tightly in order to get this exact circumference). The ring, being of the height of the crown which was broken off, was slightly contoured into shape by bending-forceps, was placed in position and pressed down around the remnants of the root quite forcibly, and the mouth having been kept dry by cotton rolls and napkin, the ring was filled with amalgam, which engaged with the threads of the screws on the one side and the contouring of the upper part of the ring on the other, and made once more a solid tooth, with cusps and indentations very much like nature in shape. (Fig. 10.) If the opposing

Fig. 8.

Fig. 9.

Fig. 10.

teeth can be bitten into this amalgam, so as to get the exact length and shape desired, and the surface be then finished by absorbing all surplus mercury by gold pellets, as before mentioned, a frosted surface is left, which is very little noticeable in the mouth. The model now exhibited shows the operation when completed.

The last thing to which I will call the attention of the Institute is a method of treating an abscessed tooth, which it is necessary to fill without much preliminary treatment. A patient was obliged to sail for Europe last Saturday. In order to save her from being troubled with a tooth so hastily treated, I placed a piece of tapered copper wire in each root. I then filled the cavity with gutta-percha, and after the filling was completed withdrew the wires, thus leaving a small opening into each root to allow the escape of any accumulations, gaseous or fluid, which might subsequently form.

*Dr. Robinson.*—I should think the gutta-percha fillings would pack down and close these openings up.

*Dr. Bogue.*—I trust eventually that it will, but they will probably remain during the week that the lady is crossing, especially as the orifices are on the buccal side of the tooth.

*Dr. Chas. O. Kimball.*—I wish to offer the especial thanks of the Executive Committee to Dr. Bogue for bringing these three cases before us to-night. I wish, speaking again for the Executive Committee, that the members would make it a habit to come to every meeting with something of practical utility. It is these things that are helpful to us and, through our report, helpful to the profession at large.

*The President.*—The invitation of the Executive Committee will, we hope, be borne in mind, and that members will come laden with ideas of a practical nature.

*Dr. Gillett.*—Regarding the method explained by Dr. Bogue, of leaving a small vent in a gutta-percha filling, for many years it has been my habit, when dressing doubtful pulpless teeth, to puncture the temporary stopping with a small point. This small opening provides an effective vent, but is not easily penetrated by the fluids of the mouth.

Dr. Kimball read a review of Dr. Talbot's paper by Dr. Newkirk.

### A SHORT REVIEW.

BY GARRETT NEWKIRK, M.D., LOS ANGELES, CAL.

Readers of the *Dental Cosmos* for the last ten years may remember a review published in the December number, 1892, of a series of papers entitled "A Study of the Degeneracy of the Jaws of the Human Race," by Dr. E. S. Talbot.

After nine years the present writer feels impelled once more to review a paper of similar tenor, read before the Chicago Medical Society, February 13, 1901, and published in the INTERNATIONAL DENTAL JOURNAL for the following November. The title of the paper is "Interstitial Gingivitis as a Prominent Obvious Early Symptom of Autointoxication and Drug Poisoning."

The author will pardon me, I hope, for saying that, while the title represents undoubtedly much study, the paper as a whole and in detail seems to have been hastily written and put forth without careful consideration of the statements contained.

To begin with a minor criticism, it seems to this writer that the term interstitial gingivitis is not as a whole desirable. Gingivitis is inflammation of the gingiva or gum. Interstitial gingivitis, therefore, is inflammation of the interstitial gum,—*i.e.*, between the teeth. This implies the existence of a gingivitis strictly localized, leaving the lingual, labial, or buccal parts unaffected, a condition rarely found.

"Autointoxication" is perhaps allowable as a medical term. It means, if anything, just this: a poisoning of the body by its own waste products. The idea is old, but the word has the disadvantage that it calls for a special definition of the popular word, intoxicate; and certainly it is better to avoid all needless confusion of the President's English.

Coming to the article itself, one has to admit that it contains here and there statements of truth which no one would question as to the results that follow from faulty elimination. Along with these, however, are others, some half-truths perhaps, others wide of the mark or distinctly erroneous. It does not matter so much when faulty statements occur in the writings of an inconspicuous man, but in those of an author of text-books, who has been a recognized leader and teacher for many years, it does matter, and that seriously. Utterances coming from such a source are accepted by many without question, especially among younger readers, and, if wrong, become in their thinking as tares among wheat. In the first paragraph of the paper we find the following:

"The sweat-glands perform their function normally in the summer, but with the first breath of cool weather the glands contract and the liver and kidneys are forced to perform the work of the skin. Autointoxication takes place. The skin of the fingers begins to peel and itching with eruption results."

Verily, this is not in accord with the experience of people generally in any latitude where seasons come and go. Even in Southern California we can hardly escape "the first breath of cool weather," which comes down from the Sierras with murderous intent. Yet, strangely enough, we welcome it. The sense of new life, the exhilaration, may be only (auto) intoxication, but we're happy, and "peel" only our thinner underwear. But if we ever should decide to have an eruption on the first breath of Boreas, desquamation would follow and not precede the other conditions.

Next: "In health, autointoxication is never noticed until after the periods of growth are complete."

We suppose the author must mean that poisoning by waste products (autointoxication) is "never noticed" till boys and girls have become fully developed men and women. If not, what does he mean? Now, if this were true, they would never get sick, and none would die except by violence. But the fact is that poisoning by waste products, as the result of faulty elimination, may occur at any time. It is doubtful if any child grows up entirely free from it.

"In health." If we have autointoxication in health, it must be a normal condition and not pathological.

"Foods taken into the system are appropriated up to this period." What period? Periods of growth?

And further: "The amount of food required depends upon waste and repair. This depends to a great extent on the avocation of the person. The older the person, the more effete matter needs removal."

About half true this. He means vocation, not "avocation." A man's vocation is his regular work. His avocation is something aside, like the gardening of a doctor, or the honest toil of a walking delegate. It is not necessarily true that "the older the person, the more effete matter needs removal." The reverse may be and usually is true. It is the instinct of man to conserve his strength, to move more deliberately, to undertake less, as age advances. The young man runs for the train, perspires, with a flushed face and palpitating heart. His father knows better, starts earlier, or waits for the next train, thereby keeping much fibre from becoming "effete."

Speaking of the first alveolar process, the writer says,—

"When these [the deciduous] teeth are lost the process absorbs" [meaning is absorbed], "but it reappears on eruption of the permanent teeth."

Now, the truth is that the process develops, expands, to accommodate the incoming guest, and is absorbed only as to the doorway by which he enters, the way by which the other departs. There is simply a new accommodation of bone. If a deciduous tooth be lost and the process *is* fully absorbed, the indication is positive that the permanent tooth is missing.

"The alveolar process simply holds the teeth in place while they are being used for mastication." May we not add, between times?

The following statements are so peculiar, both in matter and construction, that I beg leave to use italics:

"Absorption of the alveolar *process* is an inflammatory *process*. I have entitled this inflammation interstitial gingivitis, . . . *therefore,* the alveolar process as well as the gum tissue is involved."

Why "therefore"? Is the *process* "involved" because the "inflammatory process" has been named by him, or because "absorption of the alveolar *process* is an inflammatory *process*"? Or is it because the process proceeds by some unknown process?

To the author's statement, that "absorption of the alveolar process is an inflammatory process," we demur. One may lose a portion or all of an alveolar process without pain, increase of temperature, redness, or swelling. It is no more inflammatory than absorption of the deciduous teeth, or the substitution of bone for cartilage. The author has just stated, only two paragraphs distant, that it is "easily absorbed" and "the most transitory . . . of any structure of the body." How then is "absorption of the alveolar process" an "inflammatory process," which "I have entitled interstitial gingivitis"?

Immediately following in this *procession,* we come upon an indictment of modern dentistry which would certainly be remanded by the court to any lawyer for correction of terms.

"Modern dentistry is doing most to produce local irritations resulting in predisposing causes: the application of the rubber dam, clamps, wedging of teeth, correcting irregularities, sharp edges of decayed or filled teeth, crown- and bridge-work, artificial teeth, more particularly ill-fitting plates, over-stimulation in the use of toothpicks, injuries, tartar, accumulation and decomposition of food, tobacco, and everything of a foreign nature that will produce irritation."

Is this a right use of terms? What is a predisposing cause? Does the immediate or active cause precede the predisposing? Not since the first dictionary, surely. It is true, no doubt, that in modern dentistry specialties have developed with much division of labor and many added responsibilities, but is it fair to include among these the effects of tobacco, toothpicks, tartar, fermentation of food, and "everything of a foreign nature that will produce irritation"?

"Local causes, which are easily recognized and can be handled only by a dentist." Do we "handle" causes?

It might be interesting to know how many readers and hearers of the paper had any definite idea of the meaning of the following sentences: "Interstitial gingivitis produces four forms of bone absorption,—lacunar, or osteoclast, halisteresis, Volkmann's perforating canal, and osteomalacia, or senile absorption. Halisteresis and Volkmann's perforating canal absorption are naturally the most common, since they are directly due to the inflammatory process and are likewise more rapid in their action. Lacunar or osteoclast absorption is nearly always present, but is slow. Osteomalacia or senile absorption is a natural process and attacks every individual sooner or later. Interstitial gingivitis is recognized by puffiness and bleeding of the gums."

The introduction of these for the most part unfamiliar terms, and the attempt therewith to make four divisions out of simple alveolar absorption, seem to the reviewer extremely fanciful. It may not be, but it looks like, pedantry.

Volkmann's canals are defined in a late medical dictionary as, "Passages in the subperiosteal layer of bones, connecting with the Haversian canals." The latter being given as, "Canals in the *compact structure* of bone, establishing connection between the medullary cavity and surface of the bone," they can have no existence in a cancellated structure like the alveolar process. Being absent, they should not be dragged in.

"Halisteresis," according to different lexicons, means deficiency, or loss of, mineral salts. As our eminent friend G. C. might have said, "It is a condition and not a theory." Difficult to imagine, we should think, an alveolar process reduced to cartilage by the loss of its lime.

The fourth division, he says, is "*Osteomalacia,* or *senile absorption.*" We should think this a misprint were it not reiterated. And he goes on to state that it is "*a natural process*" which "attacks every individual sooner or later." By this we are told that *rickets,* a disease of early childhood, characterized by non-development of the bones, a disease of malnutrition, is one with *senile* (old age) absorption. And it "*attacks*" us all in due time.

A man who was sea-sick and sure he would die asked a friend to take charge of his remains; an hour afterwards he said, feebly, "John, you needn't mind, I don't think there'll be any remains."

Now, if we are to be "*attacked*" by a "natural process" of senile absorption, all the way from infancy to old age, the wonder is that any of us have remains.

Having shown that "autointoxication takes place" with "the first breath of cool weather;" that "in *health*" it "is never noticed until after the periods of growth are complete;" that autointoxication is inevitable in elderly people because they have more effete matter and less excreting power; that autointoxication is the cause of gingivitis; that modern dentistry is mostly responsible for the predisposing causes of gingivitis and produces four different kinds of absorption of the alveolar process, including the softening of children's bones, or senile absorption, "which is a natural process," the author adds that "Pus infection frequently takes place," and "the resulting products are taken into the stomach, producing indigestion." "Treatment consists in the patient's drinking eight or more glasses of pure water each day, in brushing the gums with a stiff tooth-brush three times a day, thereby causing them to bleed, and the employment of proper mouth-washes. Tincture of iodine should be used upon the gums and alveolar process every other day until they are restored to health," or, we presume, till the process is lost by rickets or senile absorption.

It is not easy to bring ourselves to consider such "treatment" seriously. What has become of the great mountain, autointoxication? All the remedies except water are directed to the "symptom," the local condition. Is the water given for the purpose of diluting the products of pus "which are taken into the stomach, producing indigestion," or for the dilution of pus or of gastric juice, or for an aperient or diuretic?

These cases of pus with loosening of the teeth we have been studying under various names,—pyorrhœa alveolaris, phagedenic pericementitis, etc. We have thought that out of our observation and experience we had learned a little something about them, but it would appear not. There is evidently no relation of the peridental membrane, no removal of deposits from the roots of the teeth, no special local treatment worth mentioning except by bristles and iodine. The correction of malocclusion, the support of loose teeth by mechanical means, are not to be considered.

Whenever "interstitial gingivitis is recognized by puffiness and bleeding of the gums," with "pus infection," we are to proceed with local irritation and internal hydropathic treatment. So the reviewer understands by the words written.

*Dr. Kimball.*—It seems but fair to Dr. Talbot to state that after reading his paper carefully, and then Dr. Newkirk's review, the Executive Committee recognized the fact that Dr. Talbot's was a serious paper, in which he had something to say, and that this review of Dr. Newkirk's is a pleasant way of attacking the surface peculiarities of the paper rather than its matter. They decided, however, to admit it, mainly in the hope that it might serve to make good men and true more careful about the outward form of their papers, lest that which is good in itself should be rendered, by haste or carelessness in preparation, obscure or even absurd. Accordingly this review of Dr. Newkirk's was sent to Dr. Talbot, asking him if he would not come on and discuss it, or at least write a reply to it. The committee has received the following letter from Dr. Talbot.

"January 3, 1902.

"DEAR DOCTOR,—Your letter and Dr. Newkirk's paper came duly to hand. I regret that it will be impossible for me to attend the meeting. The critique of Dr. Newkirk is one that would not be accepted by any medical body, since it displays marked ignorance of medicine, modern pathology, and philology, the two fields in which the author poses. Autointoxication, for example, is Greek, and of necessity indicates a poisoning (toxis). Good writers insist that alcoholic intoxication should be employed where Dr. Newkirk would order intoxication used alone. Since the medical profession the world over, as well as a large portion of the dental, has adopted autointoxication and employed it in the way I have done, it is senselessly futile at this late day for Dr. Newkirk to sweep (Mrs. Partington-like) the ocean of truth with his rather incoherent intellectual broom. I should also advise Dr. Newkirk to take a much-needed course under a pathologist and bacteriologist, judging from this and preceding lucubrations. A course in modern physiology is likewise imperative. Until this is done, a discussion of the " Short Review" with Dr. Newkirk would be as absurd as one with an Indian medicine man.

" Very truly,

" Eugene S. Talbot."

*The President.*—We will now have the pleasure of listening to the essayist of the evening, Dr. George S. Allan, who will read us a paper entitled " Extension for Prevention."

(For Dr. Allan's paper, see page 311.)

DISCUSSION.

*The President.*—Gentlemen, you have heard Dr. Allan's interesting paper, and I am sure we have all profited by listening to it.

*Dr. E. A. Bogue.*—Dr. Allan asks the question, " Cannot these vulnerable points be safeguarded in some other way than by cutting them away?" Yes, by cleanliness. I regard the expression " Extension for prevention" as a catch-word designed to claim and hold attention. If it results in producing a more careful preparation of approximal cavities, it will have done a real service. If it is accompanied by the injunction to carefully restore the form and size of the normal tooth (not that of a defective or malformed tooth) at the region of decay, it will do a greater service still, for contouring or restoration fillings always accomplish a certain amount of cleanliness by their insertion. Therefore, restoration of contouring should always accompany extension.

This process of extension is applicable only to a certain proportion of operations,—viz., approximal cavities, and mostly in the grinding teeth, as I shall show. But while extension to the extreme limit of disintegrated or softened tooth-substance is necessary, I do not consider extension beyond that limit desirable in cases where the structure of the teeth is good, and cleanliness may be expected. For example, I have to-day seen seven fillings inserted by myself when I was a youngster, all of them in good order; four of the seven are approximal cavities, yet in none of them was extension practised beyond the line of decay, and in none of them do the cavities go rootward beyond the enamel margin. Cleanliness has always been inculcated, and been practised by the patient.

Another patient, about twenty-four years of age, was in yesterday, one of a family of four who have been all their lives in my hands, as have their father and his father's family of six children. Contouring and cleanliness have always been practised in these families, but not extension of cavities into sound territory. Yet none of the children, save one of these last two generations, has ever lost a tooth or suffered from toothache while under my care. This one was a lady, older than myself, who suffered from pyorrhœa before I ever saw her. After such an experience, I filled the young lady's bicuspids after thorough wedging with contour or restoration fillings that do not show.

Dr. Black tells us that "extension for prevention is extension of the enamel margin, of a cavity of decay, from a line of greater liability to a line of lesser liability." Or, to change the phrase, it is "to cut the enamel margins from lines that are *not* self-cleansing, to lines that are self-cleansing."

Now, gentlemen, there are comparatively few patients who come to us possessing thirty-two teeth, and if they do not possess thirty-two teeth, or at least twenty-eight, allowing that the wisdom-teeth have not yet erupted, we need not expect any of the lines of those teeth to be self-cleansing, excepting the rounded or tuberous portions of the teeth where the enamel is thickest. The reason is two-fold: First, extractions of teeth here and there leave the remaining teeth in such positions that normal mastication cannot be performed; hence the friction that is presupposed in the statement by Dr. Black does not occur. Secondly, if it could and did occur, the substances generally eaten by people who come to us for services are mostly soft, well cooked, and lacking in the fibrous qualities that necessitate mastication and insalivation. The comparative cleanliness that would result from such a degree of friction as ought to attend the process of mastication is lacking. We therefore supplement it with the use of the brush.

Now for some examples: Two superior central incisors filled in approximal surfaces, both mesial and distal, probably with soft foil, and finished with flat or nearly flat surfaces. Attention was called to them by a minute examination, which resulted in wedging the teeth apart sufficiently to make a thorough exploration as well as to do what was required. The fillings were inserted twenty-five or thirty years ago, probably by Dr. Gunning, who in his day had scarcely a superior. Both fillings were found thoroughly compacted, smoothly polished, and in remarkably good condition considering the lapse of time. At the cervical margins of each of the two central fillings was a slight line of discoloraation, which proved to be decay at the precise point where fluids, by capillary attraction, would lodge and remain.

A gentleman, the editor of a dental journal, quite lately has asserted that teeth are not more liable to decay just above the point of contact, but just *at* the point of contact. These two teeth are decayed just above. My own observations go to show that capillary attraction has much to do with drawing and keeping deleterious fluids in contact with those portions of approximal surfaces just removed from the point of friction, which is the point of contact. Had the cavities in these teeth been extended twenty-five years ago and then been filled just as they *were* filled, there is no question in my own mind but that we should have had cavities at this time occupying exactly the same relative positions as did these. If, however, these smaller fillings had been slightly contoured at the time of their insertion, to make them the exact shape of the perfect natural tooth, I think there would not have been a recurrence of decay, either with or without extension, because the two bulging portions of the fillings would have come in contact sufficiently far down towards the cutting edges of the teeth to have left space above the fillings large enough to be self-cleansing.

Second case: Mr. J. W. G., two years ago, June 14, 1899, came with twenty-six approximal cavities, thirty-three in grinding surfaces, and five buccal cavities, and had his first lesson in regard to the necessity of keeping particles of food and débris of whatever kind from remaining lodged between the teeth. He is a college student, intelligent and conscientious, and has striven to keep the teeth that were repaired two years ago in good hygienic condition so far as cleanliness goes. The necks of all the grinding teeth from just above or just below the greatest tuberosity to the gums seem not to have been reached by brush or silk, notwithstanding these conscientious and intelligent efforts, while the portions that originally decayed (and those approximal decays took from six to twelve years to attain the size they had when I first saw them, he being eighteen years of age at that time) all seem to have been well protected by the fillings and the subsequent cleansings. The first series of operations were done in June, 1899, three and a half years ago. No extension of whatever nature could reach the portions of the teeth that are now covered with that felt-like deposit that Dr. Williams describes as the accompaniment of decay.

These facts are brought forward in order that the advocates of "extension for prevention" may express themselves more accurately for the sake of those who might otherwise take only a partial view of the subject. Let us advocate the "Prevention of extension by cleanliness."

*Dr. Gillett.*—As a student, long before I ever heard that there was such a man as Dr. Black, I was taught that the ideal shape for an approximal cavity was one that should expose the edges of the cavity to natural friction, and that it was a good plan for the cervical margin of that filling to go under the gum. I was also taught that there were many instances in which that ideal could not be attained. None of the discussion that has taken place since has materially changed my conception of this subject. In many cases I extend cavities, and even small cavities, for the reason that I feel the owner of the tooth will have it longer and will find it a more useful tooth.

I doubt if the gentlemen who have been quoted to-night, with the possible exception of one (I am not sufficiently acquainted with his methods), once in a thousand times make such extension in a bicuspid as Dr. Allan drew on the board. I feel that much of the disagreement over this subject grows out of misinterpretation of the statements made by the advocates of the theory. Those who have advocated extension for prevention have had in mind certain conditions at one end of the line,—for instance, cavities in the mouths of patients who do not keep the teeth clean and who cannot be persuaded to do so,—and with these cases in mind they have sometimes made general statements to which they themselves have noted many exceptions. Others with minds concentrated upon conditions standing quite at the other end of the line have repeatedly argued as if the advocates of extension invariably practised the method. None of the publications of the so-called "Blackites" have given me any such conception of their procedures as that which seems to be in Dr. Allan's mind. I wish time allowed me to quote one paragraph from Dr. Perry's paper already referred to, but I think I can state it fairly. Dr. Perry stated that he felt great hesitation in discussing such a subject, even with time for preparation, because of the probability of misunderstandings, and suggested that if we could discuss such matters with the cases before us for actual examination and comparison, there would be many revelations as to one another's opinions. We are very prone to misunderstand written descriptions.

Dr. Allan's suggestion, his other remedy, is a very good one, but with what proportion of our patients can he apply that remedy? It depends somewhat upon conditions, but most of the men in this room have about one grade of practice. Now, I think there are very few of you who have not a certain proportion of patients whom you simply cannot get to clean their teeth. You have patients who have never cleaned their teeth properly, and who do not know what a clean tooth is. On the other hand, you have many patients who do average cleansing, and you have a few whom you are proud of because when they first came into your hands they did not know what cleansing their teeth meant, and you taught it to them; and you have done the best work you will ever do, in teaching them to properly cleanse their teeth. It is along this line that we should work, but while we are reaching out for the goal, prevention by cleanliness (which will not be reached in this generation), we have got to do something else, the best we can with the means at our command. At that Second District meeting ridicule was too often substituted for argument. It is becoming quite the thing to call some of our Western friends "tooth jewellers," but I doubt the justness of the epithet. They have certain ideas which are correct and worthy, and they are doing good with them and seem to me to state their reasons clearly. These ideas carried to extremes like other good ideas will work mischief.

I was rather interested to note that Dr. Allan first found nothing new in this method, and then he calls it an entirely new departure. The fundamental ideas of extension for prevention have been taught for at least twenty-five years. Dr. Black has systematized them, and has most ably presented the reasoning on which the practice is founded. I consider his general statements practically unimpeachable as such when considered with their context. My understanding of Dr. Black and his followers leads me to consider the position which Dr. Allan and some other critics impute to them to be much overdrawn.

Just so long as the personal equation exists in both patient and operator, will the judgment of the latter be needed to determine the amount of extension required in a given case. To lay down a *rigid* rule of practice in this matter is impossible and useless. To state a *general* rule and the reasoning on which it is founded is wise and helpful, and it is just this assembling of the best thought of the best teachers for generations into an harmonious system and clear statement of the reasons that Dr. Black has accomplished in the particular field to which to-night's discussion is limited. The working of the rule must always be modified by that judgment which can only be acquired by daily work and thought at the operating-chair, by the age of the patient, present condition of the teeth and adjacent tissues, cleanliness of patient's habits, probable influence of the operator in inducing cleanliness, periods of probable opportunity for watching results, and by many other factors.

*Dr. T. E. Weeks.*—I feel hardly equal to discussing this paper, as I have had no opportunity of seeing it; in fact, I only knew yesterday what the paper of the evening was to be about. Dr. Gillett has expressed almost all that I would say in regard to it. I was very much amused by, and at the same time appreciated very much, the retort of Dr. B. Holly Smith when he quoted Abraham Lincoln's story that a soldier's legs should be long enough to reach from his body to the ground. It seemed to me to express the situation pretty well. I was very much amused to note that several of Dr. Allan's quotations seemed to refute the point he was trying to make. The trouble is that we look at the picture from different stand-points. There is a great deal of truth in the statement made by Dr. Gillett that you here in the East are dealing with a different class of cases from those with which we in the West are dealing. Now, as a matter of fact, I do not see a great many cavities like those Dr. Allan has depicted for us on the board. Consequently, gentlemen, I think if you were to see the operations coming from my hands you would think that I was extreme.

I think if you will read the context in Dr. Johnson's book you will find that Dr. Allan hardly read enough or with the right emphasis. I know what Dr. Johnson believes, and I certainly think a different meaning was implied if not expressed in the context.

(Quotes from page 83, Dr. Johnson's book.)

I would say, gentlemen, that I am a "Blackite," but I am not an extremist; neither is Dr. Johnson, and I do not consider Dr. Black one. What we are trying to get at are the true principles that underlie the salvation of the teeth. We in the West believe in cleanliness, and we do not believe in extreme measures except when they seem to be absolutely necessary. It all becomes a study as to how much of the tooth to cut away to give the greatest strength to that portion which remains, and to give shape and contour to the resulting surface; how much to cut and how to cut to leave the enamel margin so that it will not fritter away or break down *en masse* under the strain of mastication. All must be taken into consideration. As Dr. Gillett has said, what Dr. Black would like to have credit for is the systematizing and the attempt to reduce matters to primary principles. I think there will not be much profit in these discussions until we can reach some common basis and look at the picture from the same stand-point.

*Dr. Gillett.*—I want to express my agreement with Dr. Allan's remarks regarding sloppy work. I feel that one of the things that should be impressed upon the younger practitioners is that, regardless of extension, a filling is a failure if not mechanically perfect to begin with.

*Dr. S. E. Davenport.*—It seems to me that there is one point of great importance in this question which has had too little attention, and that is the matter of contouring. If small approximal cavities are prepared without being extended much beyond the zone of actual decay,—I care little what material is used, so long as it is one that has proved to be fairly durable in that particular mouth,—care being taken to shape the fillings so that they will present but a small point of contact either with the filling in the adjoining tooth or with the tooth-structure itself, such fillings will be very durable in mouths of average cleanliness. It has seemed to me that papers and discussions upon this subject have given too little attention to this point of proper contouring.

*Dr. F. Milton Smith.*—I was delighted to hear from Dr. Weeks to the effect that we in the East have misunderstood our Western friends. I have had an opportunity to see, within the past few weeks, an example of as magnificent work as I have ever seen in my life. It came from the wild and woolly West. I wish some of us here in the East knew how to do as well. If we did we would save the teeth of our patient's better.

It seems to me that Dr. Gillett, in his short talk, has given us a good deal of what Dr. Perry calls "horse sense." We do not view these things from the same point. That is one reason why we differ so largely from one another. I find the discussion is confined almost entirely to molars and bicuspids, but as I read Dr. Black and others, they do most certainly apply the method to incisors as well. I should like to read one or two quotations from Dr. Black.

(Quotations read from Dr. Black's book, pages 92 and 95, to the effect that margins of cavities must be self-cleansing or covered by gum-tissue.)

By "self-cleansing," then, he means that they must reach a point where they either can be cleansed by the brush or else be covered by gum-tissue. If this is a fair statement, and if Dr. Black means what he says, I most certainly do take issue with him, for I do not believe that in anything like all cases should the gingival margin be covered by gum-tissue. Referring to the articles in the *Items* it seems to me that any one who wants to get at the base of the subject ought not to fail to read the May number, 1891. It is full of practical thoughts and suggestions upon this point, and it makes one glad he is alive to know that there are so many men who are full of good thoughts upon this subject. Two of these articles have been especially useful to me,—the one by Dr. Darby, of Philadelphia, and that by Dr. Perry, of New York. They put the matter in a light exactly as I like it. Dr. Darby does not believe that we are justified in such wholesale cutting and the sacrificing of so much tooth-structure, and Dr. Perry says that, even admitting that these radical operations are more lasting, they are not always called for. He does not think that we are always called upon to do perfect work in the mouths of our patients, and very often work less perfect is more satisfactory. It seems to me that that pretty nearly covers the ground.

*Dr. Weeks.*—I simply wish to state that, so far as I can remember, I got my first ideas in regard to this matter from a gentleman who worked with Dr. Perry and had Dr. Perry's ideas.

So you see my original ideas in this direction came from the East. I do not forget Dr. Perry and those other noble men who contributed so much to our progress.

*Dr. Allan.*—First let me say, in reply to Dr. Weeks, that no man honors Dr. Black more than I do, and I think I am as much of a Blackite as Dr. Weeks is. I stand by him and the work he has done, especially in the systematizing of these methods. I cannot, though, accept his theories in their entirety, much less his principles and methods of practice based thereon. This apologizing and explaining things, trying to make radicalism based on one idea (theory or dogma, call it what you will) harmonize with conservative thought and method based on long experience and thoughtful attention to all laws governing the subject, seems to me most unwise and lacking in force of application. Western men, as a rule, are honest in their convictions and know just what they say, and they do not readily apologize; much less will they thank any one who makes an apology for them. In this extension for prevention theory they know exactly what they mean, and expect to stand by their colors. They fully believe we are behind the times, and that they are up to date and a few years ahead.

Regarding the applicability of the system to others than molars and bicuspids, the system is applied to front teeth in exactly the same way.

As to not understanding Western gentlemen, I claim that, within reasonable limits, I can understand the English language.

*Dr. Weeks.*—We do not speak it out West.

*Dr. Allan.*—As I read Dr. Black's articles, and as I look at his diagrams and drawings showing how much tooth-structure is taken away, I tell you it amounts to just what I have stated, and I will not take back one thing I have said. Either they do not speak the English language or they do not know how to draw, or both.

Now, when we take an instrument to examine a filling, and find that the decay around that filling extends into the tooth beside the filling so that you can place the instrument to the bottom of the original cavity beside the filling, I tell you it means a poor filling. But this is not the same when the decay extends for some distance away from the margins of the filling, and when your instrument comes to the filling it will not go in beside it. This radical system is advocated by some of the text-books, and especially in the treatise by Dr. Johnson, whom I consider to be a most extreme man. When a man virtually tells all the rest of the profession that they are twenty years behind the times, the ninety-nine fellows so stigmatized feel like replying just a little to this. I do not think we are behind the times, but that we can take in new ideas just as quickly as any men, West or North or South.

*Dr. R. H. M. Dawbarn.*—I do not wish to make a speech, but simply a little announcement,—namely, that for the first time in the history of this island the dental profession has been recognized as it should be recognized, as a specialty of medicine in the largest hospital in this city, the City Hospital. As you already know, a representative of this Institute and one from the First District Dental Society were appointed at my request to represent the profession at the City Hospital, and they have done faithful work for a year past, but they were not given a seat nor a vote in the board. They have occupied the same position as the pathologist. But at the last annual meeting, that was held this week, I brought the matter up and succeeded in carrying it through, so that now dentists will have a seat and a vote in the board exactly as other specialties. I am very glad, Mr. President, of having had this opportunity of bringing this matter before the Institute.

Adjourned.

FRED. L. BOGUE, M.D., D.D.S.,
*Editor The New York Institute of Stomatology.*

## Figures and Tables

**Fig. 1. f1:**
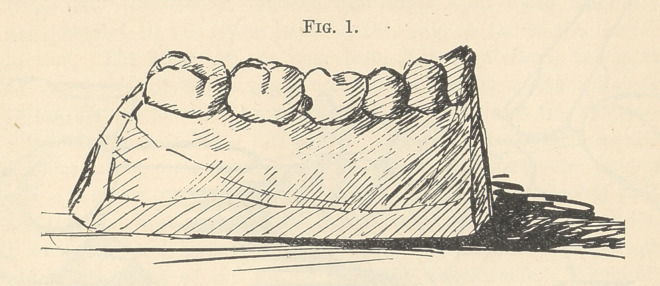


**Fig. 2. f2:**
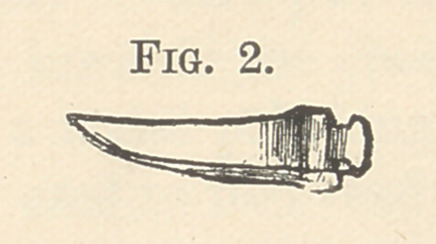


**Fig. 3. f3:**
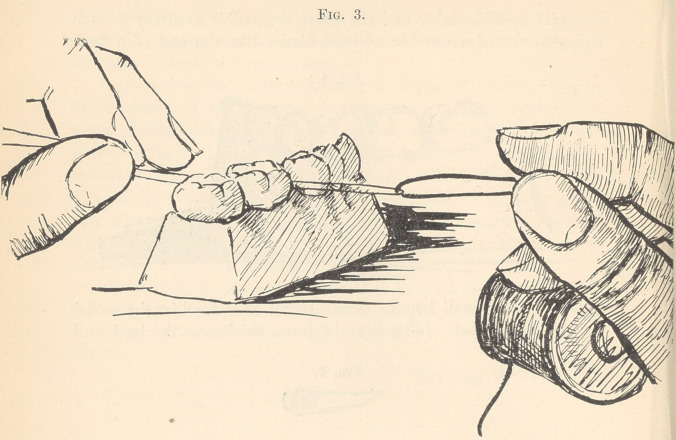


**Fig. 4. f4:**
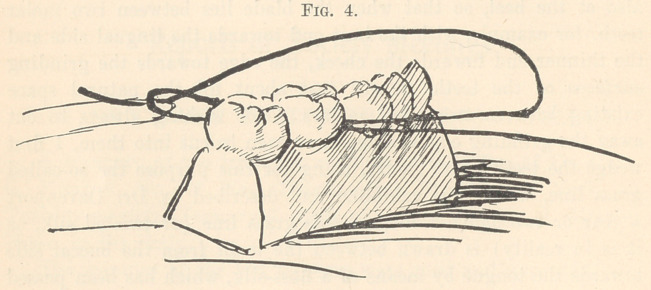


**Fig. 5. f5:**
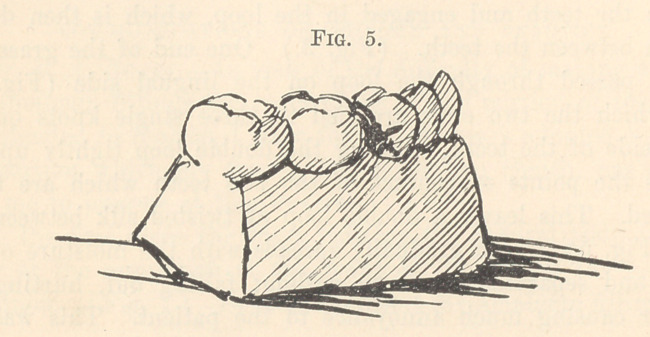


**Fig. 6. f6:**
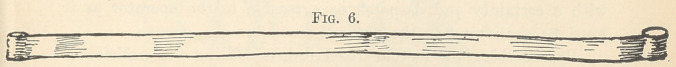


**Fig. 7. f7:**
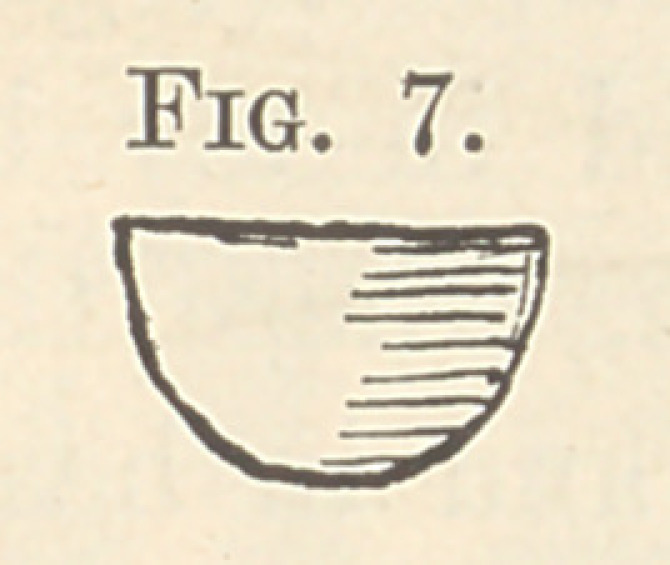


**Fig. 8. f8:**
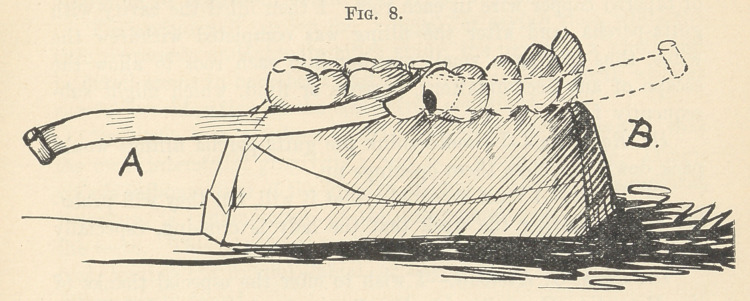


**Fig. 9. f9:**
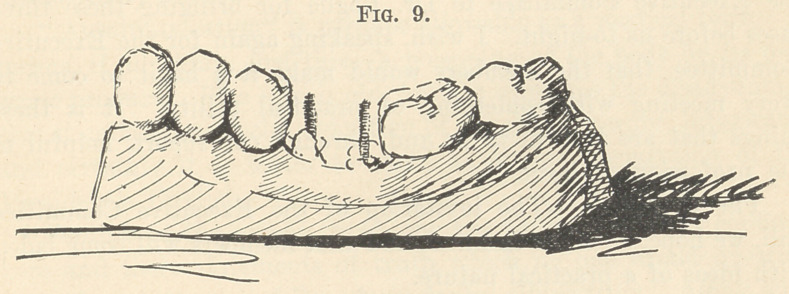


**Fig. 10. f10:**